# *Aspergillus* Oxylipin Signaling and Quorum Sensing Pathways Depend on G Protein-Coupled Receptors 

**DOI:** 10.3390/toxins4090695

**Published:** 2012-09-18

**Authors:** Katharyn J. Affeldt, Marion Brodhagen, Nancy P. Keller

**Affiliations:** 1 Department of Bacteriology and Department of Medical Microbiology and Immunology, 1550 Linden Drive, Madison, WI 53706, USA; Email: kjschmitt2@wisc.edu; 2 Department of Biology, Western Washington University, 516 High Street, Bellingham, WA 98225, USA; Email: Marion.Brodhagen@wwu.edu

**Keywords:** aflatoxin, *Aspergillus*, oxylipin, G protein-coupled receptor (GPCR), quorum sensing, sclerotia

## Abstract

Oxylipins regulate *Aspergillus* development and mycotoxin production and are also involved in *Aspergillus* quorum sensing mechanisms. Despite extensive knowledge of how these oxylipins are synthesized and what processes they regulate, nothing is known about how these signals are detected and transmitted by the fungus. G protein-coupled receptors (GPCR) have been speculated to be involved as they are known oxylipin receptors in mammals, and many putative GPCRs have been identified in the Aspergilli. Here, we present evidence that oxylipins stimulate a burst in cAMP in *A. nidulans*, and that loss of an *A. nidulans* GPCR, *gprD*, prevents this cAMP accumulation. *A. flavus* undergoes an oxylipin-mediated developmental shift when grown at different densities, and this regulates spore, sclerotial and aflatoxin production. *A. flavus* encodes two putative GprD homologs, GprC and GprD, and we demonstrate here that they are required to transition to a high-density development state, as well as to respond to spent medium of a high-density culture. The finding of GPCRs that regulate production of survival structures (sclerotia), inoculum (spores) and aflatoxin holds promise for future development of anti-fungal therapeutics.

## 1. Introduction

*Aspergillus flavus* is a soil-borne pathogen that infects a variety of food and feed crops including maize, peanuts, cottonseed, and tree nuts, both pre- and post-harvest. *A. flavus* produces aflatoxin (AF), the most potent natural carcinogen known [1], which causes fatal aflatoxicosis at high doses. At low doses, AF is still very dangerous as it causes a critical mutation in the tumor suppressor protein p53, leading to hepatocellular carcinoma, or liver cancer [2,3]. Beyond the health implications, *A. flavus* outbreaks occur across the globe, causing devastating losses to local food supplies and local economies in the billion-dollar range worldwide [4,5]. To develop effective means of combatting this pathogen, a deeper understanding is needed of the basic developmental pathways that lead to AF biosynthesis and the persistence of the fungus through asexual spores and recalcitrant overwintering structures, called sclerotia. Spores are the initial inoculum of the fungus, and sclerotia allow the fungus to survive in the soil over harsh environments, such as drought and cold temperatures.

One of the primary signals for *Aspergillus* spore and sclerotia development are oxylipins (oxygenated polyunsaturated fatty acids), which act as autocrine and paracrine mediators in eukaryotic organisms [6]. In addition to their endogenous functions, oxylipins can also engage in inter-organismal signaling across multiple phyla [7,8,9] including *Aspergillus*/seed cross-talk [10,11,12,13]. Oxylipins are made by oxygenase enzymes, found throughout the tree of life, that incorporate oxygen into a fatty acid backbone. Study of these enzymes in the Aspergilli and other fungi has revealed their importance in fungal development and mycotoxin production [14,15,16,17,18]. 

*Aspergillus nidulans* is often used as a model organism to elucidate developmental processes in other filamentous fungi, including *A. flavus.* In *A. nidulans,* three dioxygenase enzymes (PpoA, PpoB, and PpoC) contribute to oxylipin production [19,20,21]. Deletion of *ppo* genes affects the gene transcription and production of at least two secondary metabolites in *A. nidulans*: the carcinogen sterigmatocystin (the penultimate precursor to AF), and the antibiotic penicillin [11]. Simultaneous loss of all three *ppo* genes shifts development from asexual to sexual reproduction [21]. Loss of some of these genes in *A. fumigatus* leads to both altered spore development and toxin synthesis that affects the outcome of disease development in murine models of aspergillosis [22,23]. In *A. flavus*, simultaneous silencing via RNAi of four homologs of the *A. nidulans* dioxygenase genes (*ppoA*, *ppoB*, *ppoC*, and *ppoD*) and one lipoxygenase gene (*loxA*) yields a shift from asexual development to the production of sclerotia (the structure in *A. flavus* analogous to sexual reproductive structures in *A. nidulans*), with an increase in AF synthesis [16,17]. Furthermore, studies with *A. flavus* mutants suggest oxylipins regulate secondary metabolism and spore development via a density-dependent mechanism resembling quorum sensing [16,17]. 

Quorum sensing (QS) is a density-dependent phenomenon that leads to a coordinated response from the population, such as biofilm formation by *Psuedomonas aeruginosa* and bioluminescence by *Vibrio fischeri*. As the population grows, cells secrete an inducer molecule until it surpasses a certain threshold, activates its receptor, and initiates gene transcription. Although once thought to exist only in bacteria, QS systems are now well established in fungi [24,25]. Often lipid moieties including oxylipins are key to QS in fungi [26]. For example, *A. flavus* undergoes a density-dependent shift in which, at low population densities, production of conidia is low, while production of sclerotia and AF is high. At high density the inverse is seen: production of sclerotia and AF is low, while conidiation is increased. *A. flavus* oxygenase mutants do not display this same pattern of development, suggesting that oxylipins are important in orchestrating this phenomenon [16,17]. Recent work with *A. nidulans* also supports an oxylipin-driven quorum sensing system that impacts spore germination [27].

The hypothesis that oxylipins—both *Aspergillus* and plant derived—could be involved in quorum sensing is also supported by several chemical induction studies. Notably, exposure to the exogenous seed oxylipins 9(*S*)-hydroperoxyoctadecadienoic (9(*S*)-HpODE) acid and 13(*S*)-hydroperoxyoctadecadienoic acid (13(*S*)-HpODE) stimulate sporulation and respectively enhance or inhibit sterigmatocystin/AF synthesis in *A. nidulans*, *A. flavus*, and *A. parasiticus* [28,29]. Furthermore, 13(*S*)-HpODE has been shown to inhibit sclerotial production by certain strains of *A. flavus* [29]. Exogenous applications of mixtures of native *Aspergillus* oxylipins also shift the balance of asexual to sexual/sclerotial production in all three *Aspergillus* species [29].

Despite this extensive evidence for oxylipins as drivers of *Aspergillus* development and AF production, nothing is known about how fungi perceive oxylipins. In mammals, oxylipins are recognized by G protein-coupled receptors (GPCRs). For example, GPCRs are receptors for both prostaglandins (cyclooxygenase-generated oxylipins) and leukotrienes (lipoxygenase-generated oxylipins) involved in inflammation and asthma progression [30,31]. Mammalian cells also generate some of the same linoleic acid-derived oxylipins as plant cells, including the AF-inducing metabolite 9(*S*)-HpODE, its structural analog 9-hydroxyoctadecadienoic acid (9(*S*)-HODE), 13(*S*)-HpODE, and 13(*S*)-HODE [32,33]. These oxylipins are recognized by the human GPCR G2A [34,35,36]. Although genome analysis has identified many fungal GPCRs [37,38,39], the only currently known GPCR ligands are sugars, amino acids, peptide pheromones and photons [40,41]. Here we investigate the hypothesis that oxylipins are perceived by fungal GPCRs. We find that several oxylipins stimulate a burst in cAMP, a downstream event of GPCR activation, in *A. nidulans*, and this may be mediated at least in part by the GPCR GprD. We further report that the two putative homologs of GprD in *A. flavus*, GprC and GprD, are required for proper density-dependent development. When depleted of both receptors, the fungus is locked in a low density state, even when grown at high density, and does not respond to spent medium of a high density culture. 

## 2. Results and Discussion

### 2.1. Oxylipins Exhibit Link to GPCR Signaling

#### 2.1.1. Oxylipins Induce a cAMP Burst in *Aspergillus nidulans*

**Figure 1:**
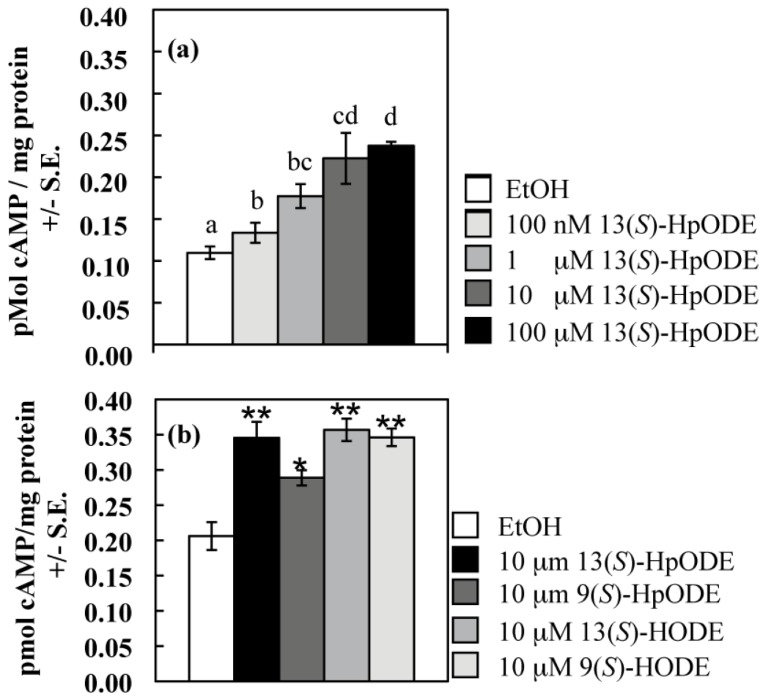
(**a**) Samples were treated with EtOH (control) or an equivalent volume of 13(*S*)-HpODE dissolved in EtOH to achieve the final sample concentrations listed; (**b**) Samples were treated with EtOH (control) or an equivalent volume of pure oxylipin dissolved in EtOH to achieve a final sample concentration of 10 μM. For both (a) and (b), tissues were harvested as described, and cAMP concentrations were measured. Differing letters above bars in (**a**) denote treatments significantly different from one another (*p*≤ 0.05; one-tailed paired Student’s *T*-test). Differences from the EtOH control in (**b**) are denoted as follows: *p < 0.05; **p < 0.01, determined by one-tailed paired Student’s *T*-tests.

A hallmark of G protein signaling is alteration of cAMP levels; indeed, binding of 9(*S*)-HODE to the mammalian GPCR, G2A, inhibits cAMP accumulation [35]. Because the role of cAMP in development had already been described for the genetic model *A. nidulans* [42,43,44,45,46], the first *Aspergillus* species studied for oxylipin developmental effects [19], we measured cAMP levels in tissues of this fungus exposed to pure plant oxylipins. We first examined the wild type response to increasing concentrations of 13(*S*)-HpODE and found the cAMP burst to be released in a dose-dependent manner. Fungal tissues were treated with 12.5 μL EtOH containing increasing concentrations of 13(*S*)-HpODE with a resulting increasing production of cAMP (all *p* < 0.05; Figure 1a). Previously, individual oxylipin species were measured in homogenized *A. nidulans* tissues at approximately 30 to 110 nmol/g dry weight [12]. In the current study, assuming a water content of 70% in fungal tissues, 33 nanomoles oxylipin were added per gram dry weight when oxylipins were added at 100 nM and correspondingly more at higher concentrations (Figure 1a). At 10 μM, the cAMP response appeared to be at saturation (as also seen for 9(*S*)-HODE perceived by G2A [35]), and increasing from 10 μM to 100 μM 13(*S*)-HpODE did not cause a significant difference in cAMP response (*p* = 0.6), so oxylipins were applied at 10 μM in subsequent experiments.

The fungus was then examined for response to four oxylipins: 13(*S*)-HpODE, 9(*S*)-HpODE and their hydroxy derivatives, all of which activated the mammalian GPCR, G2A [35]. These four metabolites are generated by both plant and mammalian lipoxygenases [32,33,47,48]. As seen in Figure 1b, wild type *A. nidulans* produced more cAMP after a twenty second exposure to each of these oxylipins than after a twenty second exposure to the same volume of EtOH carrier (all *p* < 0.05). 9(*S*)-HpODE caused the smallest response; the other three oxylipins caused a nearly identical increase in cAMP. The act of grinding effectively spreads cAMP uniformly throughout tissues; however, local responses to oxylipins at the cellular level may have created much higher gradients of cAMP than we could measure with our methods. 

**Figure S1:**
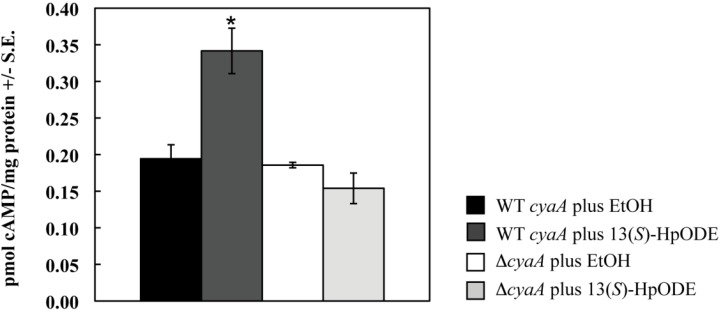
Samples were treated with EtOH (control) or 10 μM 13(*S*)-HpODE in EtOH. Tissues were harvested as described, and cAMP concentrations were measured. Differences from the EtOH control are denoted as follows: *p < 0.05, determined by a one-tailed paired Student’s *T*-test.

As a final control to confirm accuracy of our methodology, we assessed an adenylate cyclase mutant (*∆cyaA*, [44]) and its parental wild type *cyaA* strain for their responses to 13(*S*)-HpODE. Adenylate cyclase is required for activation of cAMP release. As expected, addition of 13(*S*)-HpODE to the wild type increased cAMP production 1.8 fold over the EtOH control (*p* = 0.05). However, there was no significant difference between samples of the *∆cyaA* mutant treated with EtOH or 13(*S*)-HpODE (*p* = 1.4; Figure S1).

#### 2.1.2. An *A. nidulans ∆gprD* Mutant Does Not Accumulate cAMP in Response to Oxylipins

Having established the cAMP response to oxylipins, we next assessed a series of GPCR mutants for alterations in oxylipin response. As a preliminary screen, we first assessed the ability of the ten GPCR deletion mutants that were constructed at the time of this study (*∆gprA*, *∆gprB*, *∆gprC*, *∆gprD*, *∆gprE*, *∆gprF*, *∆gprG*, *∆gprH*, *∆gprI*,and *∆gprK*, Table 1) to respond to polyunsaturated fatty acid (linoleic and arachidonic acids) saturated discs by hyper-conidiation. This method provides a rapid and inexpensive screen for lack of response to oxylipins [29]. Of the ten GPCR mutants, *∆gprA* and *∆gprD* mutants did not exhibit hyper-conidiation when exposed to 0.5 and 1 mg fatty acid-soaked discs, with *∆gprD* showing the greatest defect (data not shown). 

**Table 1:** All strains used in this study and their genotypes.

Species	Strain	Genotype	Source
*A. nidulans*	RDIT9.32	Wild type ( *veA*)	[19]
*A. nidulans*	RKIS1	*pabaA1*; *yA2*; *veA1*	[44]
*A. nidulans*	RKIS47.1	*pabaA1*; *yA2*; *∆cyaA::pyrG*; *veA1*	[44]
*A. nidulans*	RDWC2.2	*∆gprA::argB*; *veA*	This study
*A. nidulans*	RDWC7.2	*∆gprB::argB*; *veA*	This study
*A. nidulans*	RDWC4.5	*∆gprC::argB*; *veA*	This study
*A. nidulans*	RDWC5.4	*∆gprD::argB*; *veA*	This study
*A. nidulans*	rJH12.9	*∆gprE::argB*; *veA*	J.-H. Yu, UW-Madison
*A. nidulans*	rJH21.1	*∆gprF::argB*; *veA*	J.-H. Yu, UW-Madison
*A. nidulans*	RDWC1.2	*∆gprG::argB*; *veA*	This study
*A. nidulans*	rJH34.12	*∆gprH::argB*; *veA*	J.-H. Yu, UW-Madison
*A. nidulans*	rJH42.14	*∆gprI::argB*; *veA*	J.-H. Yu, UW-Madison
*A. nidulans*	RDWC6.5	*∆gprK::argB*; *veA*	This study
*A. nidulans*	TJAR39	*yA2*; *pabaA1*; *∆gprA::argB*; *argB2*; *veA1*	[49]
*A. nidulans*	TKH3.33	*metG1*; *biA1*; *∆gprB::argB*; *argB2*; *veA1*	J.-H. Yu, UW-Madison
*A. nidulans*	RKH68.8	*yA2*; *pabaA1*; *∆gprC::argB*; *argB2*; *veA1*	J.-H. Yu, UW-Madison
*A. nidulans*	RKH57.25	*biA1*; *∆gprD::argB*; *argB2*; *veA1*	[50]
*A. nidulans*	RKH75.2	*yA2*; *metG1*; *pabaA1*; *biA1*; *∆gprG::argB*; *veA1*	J.-H. Yu, UW-Madison
*A. nidulans*	TJAR13	*∆gprK::argB*; *argB2*; *veA1*	J.-H. Yu, UW-Madison
*A. flavus*	NRRL3357	Wild type	[51]
*A. flavus*	3357-5	*pyrG^-^*	[51]
*A. flavus*	TKJA10.1	*∆gprC::pyrG*; *pyrG^-^*	This study
*A. flavus*	TKJA8.1	*∆gprD::pyrG*; *pyrG^-^*	This study
*A. flavus*	TKJA14.2	*gprC*; * gprD IRT::pyrG*; *pyrG^-^*	This study

**Figure 2:**
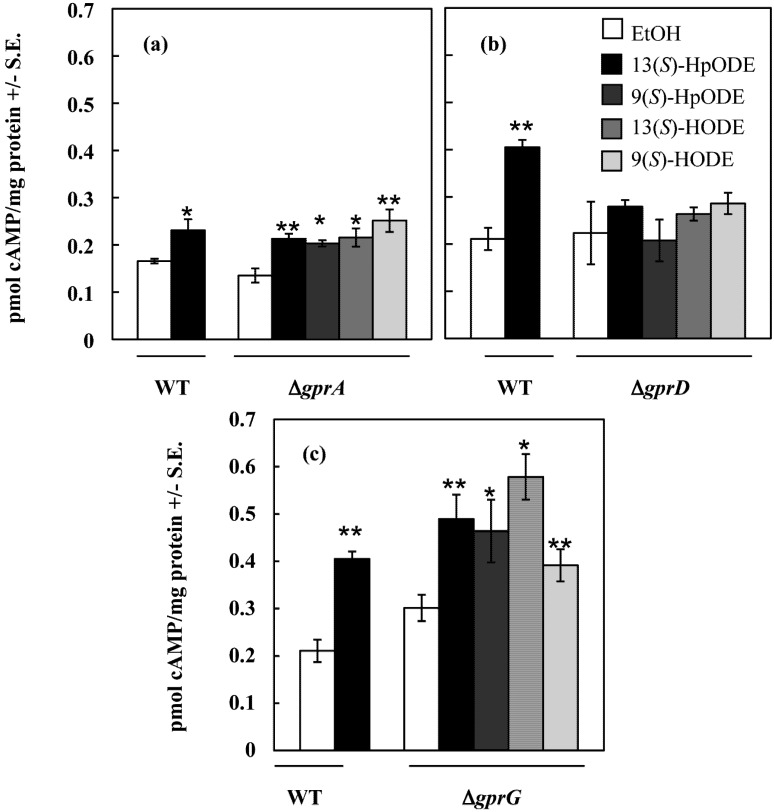
(**a**) *A. nidulans* wild type and *∆gprA* were treated with EtOH (control) or 10 μM oxylipin in EtOH, tissues were harvested, and cAMP concentrations were measured; (**b**) *A. nidulans* wild type and *∆gprD* were treated with EtOH (control) or 10 μM oxylipin in EtOH, tissues were harvested, and cAMP concentrations were measured; (**c**) *A. nidulans* wild type and *∆gprG* were treated with EtOH (control) or 10 μM oxylipin in EtOH, tissues were harvested, and cAMP concentrations were measured. Differences from the EtOH control in (a), (b), and (c) are denoted as follows: *p < 0.05; **p < 0.01, determined by one-tailed paired Student’s *T*-tests.

To better assess the possibility that GprA or GprD might be involved in oxylipin perception, *A. nidulans* ∆*gprA* and ∆*gprD* strains, as well as ∆*gprG,* a randomly chosen GPCR deletion mutant exhibiting the wild type conidiation response to linoleic acid, were then assessed for their cAMP response to four pure oxylipins: 13(*S*)-HpODE, 9(*S*)-HpODE, 13(*S*)-HODE and 9(*S*)-HODE. As shown in Figure 2, both *∆gprA* and *∆gprG* produced significantly more cAMP when exposed to each of the four oxylipins than when treated with an equivalent volume of EtOH (all *p* < 0.05). In contrast, the *∆gprD* mutant did not respond to any of the four oxylipins with an increase in cAMP concentrations over that of the EtOH control (all *p* > 0.1). 

### 2.2. Putative GprD Homologues Are Involved in Density-Dependent Development in *A. flavus*

#### 2.2.1. *A. flavus* Encodes Two Putative Homologues of *A. nidulans* GprD

**Figure S2:**
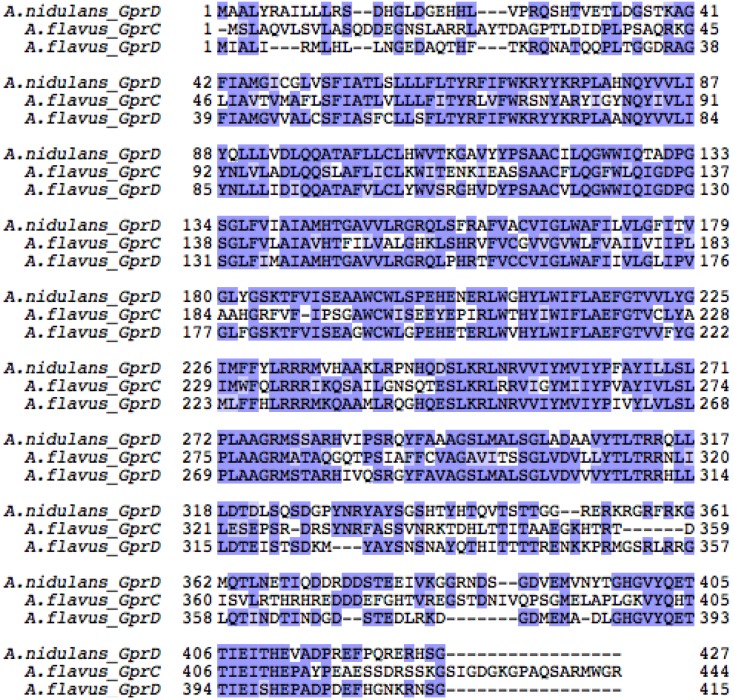
Amino acid sequences for *A. nidulans* GprD and *A. flavus* GprC and GprD were aligned using Clustal Omega [55,56] and Jalview [57,58] with Blosum62 coloring scheme. According to this scheme, gaps are colored white, matching residues are colored dark blue, and non-matching but positively scored residues are colored light blue.

Because of the growth and sporulation defects of the *A. nidulans ∆gprD* strain [50] and it not being a pathogen of note, we followed up with physiology studies with mutants of the homologous *gprD* genes in the agriculturally relevant species, *A. flavus.* As *A. nidulans* is a model organism for other filamentous fungi, including *A. flavus*, we reasoned that if GprD was involved in oxylipin perception, then it could play a role in *A. flavus* biology, as *A. flavus* oxylipin mutants are aberrant in spore and sclerotial development [16,17]. *A. nidulans* encodes at least sixteen putative GPCRs [38]. BLAST searches querying each of these GPCRs identified fifteen putative GPCRs in *A. flavus*. Of these, two appear to be highly similar to *A. nidulans* GprD. The first of these is *A. flavus* GprD (AFLA_135680), which shares 67% amino acid identity (e-value 2e-164) with *A. nidulans* GprD. The second we call GprC (AFLA_074150), and it shares 47% identity (e-value 6e-87) with *A. nidulans* GprD and 47% identity with *A. flavus* GprD (e-value 1e-119). An alignment of all three GPCRs is provided in Figure S2.

**Figure S3:**
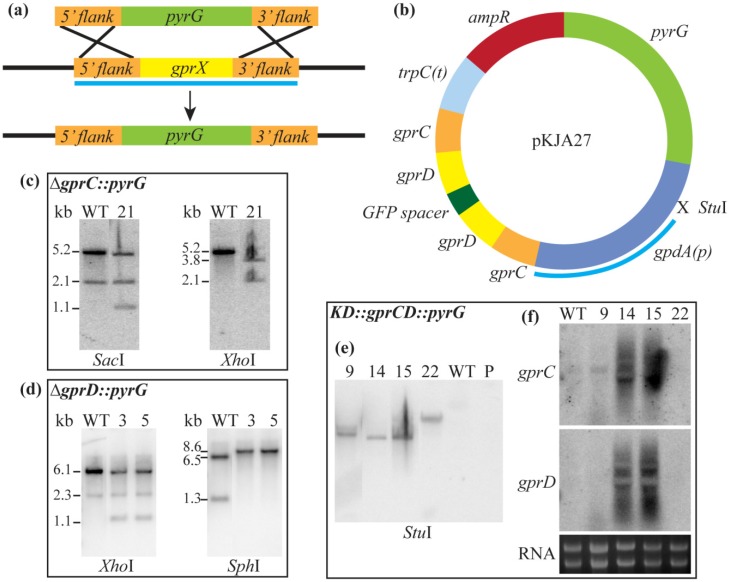
(**a**) The general scheme for deleting *A. flavus gprC* and *gprD* by replacing with *pyrG* is shown here. The light blue bar represents the region amplified for the Southern probe; (**b**) The plasmid used to deplete *A. flavus gprC* and *gprD* transcripts, pKJA27, is depicted here. The light blue bar represents the region amplified for the Southern probe; (**c**) The *∆gprC::pyrG* strain, TKJA10.1, was confirmed by Southern analysis. Genomic DNA was digested with *Sac*I (WT expected bands: 5.2 and 2.1 kb; *∆gprC* expected bands: 4.9, 2.1, and 1.1 kb) and *Xho*I (WT expected band: 5.2 kb; *∆gprC* expected bands: 3.8 and 2.1 kb); (**d**) The *∆gprD::pyrG* strain, TKJA8.1, was confirmed by Southern analysis. Genomic DNA was digested with *Xho*I (WT expected bands: 6.1 and 2.3 kb; *∆gprD* expected bands: 5.8, 2.3, and 1.1 kb) and *Sph*I (WT expected bands: 6.5, 6.1 (faint), and 1.3 kb; *∆gprD* expected bands: 8.6 and 6.1 (faint) kb); (**e**) The *KD::gprCD* strain, TKJA14.2, was confirmed by Southern analysis. The *gpdA* promoter is derived from *A. nidulans*, so the probe will only hybridize if pKJA27 is present. Genomic DNA was digested with *Stu*I (WT and parental strain 3357.5 (denoted “P”) should have no bands; transformants should have one band for each copy of the plasmid they integrated); (**f**) The *KD::gprCD* strain, TKJA14.2, was confirmed by Northern analysis. Probes within the coding regions of *gprC* and *gprD* were used, and correct transformants were identified by the smear of degraded transcripts, seen for transformants #14 and #15.

Individual deletion mutants of both *gprC* and *gprD* were created by replacing each gene with *pyrG* (Table 1, Figure S3). A third strain, *KD::gprCD* (for “knock-down of *gprC* and *gprD*”) with both genes down-regulated by RNAi [52], was also created with the thought that the two proteins may have overlapping function due to their high identity (Table 1, Figure S3). All mutants were confirmed with Southern blots, and in the case of *KD::gprCD*, Northern blots as well (Figure S3).

#### 2.2.2. *gprC* and *gprD* Mutants Exhibit Aberrant Density-Dependent Development

**Figure 3:**
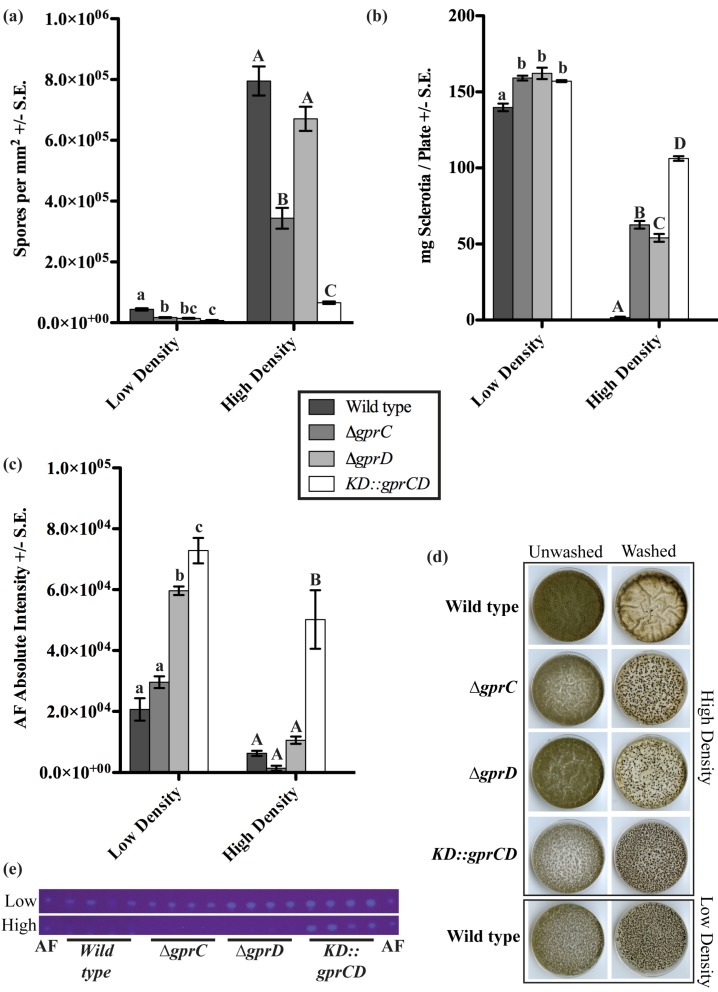
(**a**) Conidia produced by low density and high density cultures were counted; (**b**) Sclerotia were collected from low density and high density cultures, and their dry weight was measured; (**c**) Aflatoxin (AF) was extracted from low density and high density cultures, separated by thin layer chromatography (TLC), and visualized under 366-nm light. The absolute intensities of the AF spots were calculated as described in the Experimental section. For all three graphs in (a), (b), and (c), the data were analyzed using one-way ANOVA and a Tukey post-test. Different letters represent statistically significant differences (*p* < 0.05), with lowercase letters used for low-density data and uppercase letters used for high-density data; (**d**) A sample of the plates is shown, containing a full set of high-density plates (top four panels) both before (“Unwashed”) and after (“Washed”) washing off conidia. The wild type at low density (lowest panel) is also included for a point of reference; (**e**) The TLC plates for low and high density cultures are shown here. An AF standard was run on either side of the plates.

Considering the critical role of oxylipins in *A. flavus* quorum sensing development and the possibility that GprC/D could be involved in oxylipin perception, the *A. flavus* GprC and GprD mutants were examined for density dependent development. The fungi were grown at low (10^3^ spores per plate) and high (10^7^ spores per plate) densities, and production of conidia, sclerotia, and AF were measured after seven days (Figure 3). At low density, there were subtle differences between the mutants and wild type (NRRL3357), but the most striking differences were seen at high density. Here, production of conidia was high in the wild type, but it was reduced 2.3 fold in the *∆gprC* strain and was almost completely absent in the *KD::gprCD* mutant (both *p* < 0.001, Figure 3a,d). Conversely, sclerotia production was minimal in the wild type at high density. As seen in Figure 3b,d, all three mutants exhibited profound increases in sclerotia production at high density. The *∆gprC* and *∆gprD* strains produced 35.8 and 30.9 fold more sclerotia, respectively, than the wild type, while the *KD::gprCD* strain produced 60.7 fold more sclerotia than the wild type, approximately doubling what the two single mutants produced (all *p* < 0.001). AF biosynthesis was also regulated in response to population density changes, being up-regulated at low density and down-regulated at high density as previously reported [16]. The *∆gprD* and *KD::gprCD* mutants showed modest increases in AF compared to the wild type at low density. Most remarkably, the *KD::gprCD* mutant produced more AF at high density than wild type AF production at the favorable low density conditions (Figure 3e).

### 2.3. GprC and GprD Are Required for Proper Responses to Spent Media Extracts

#### 2.3.1. High Density Extracts

**Figure 4:**
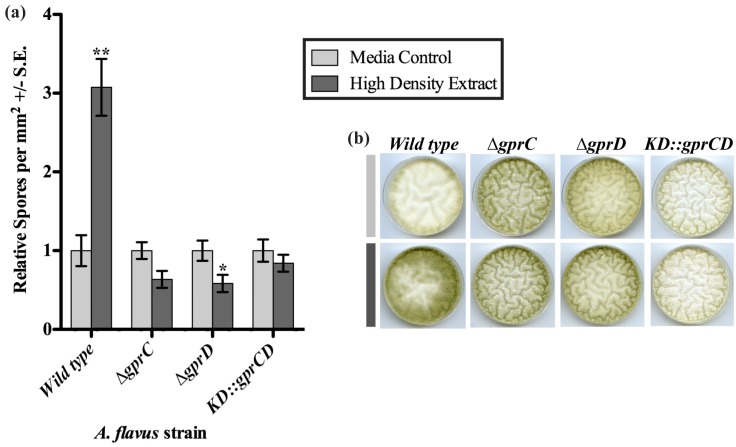
(**a**) Spores were counted from strains grown on plates containing extracts from uninoculated media (light gray bars) or spent medium of high-density wild type cultures (dark gray bars). The spore counts from plates with the media control extract were set to one, and the spore counts from the same strain exposed to the high-density extract were expressed in relation to the media control counts. Differences between the two treatments for each strain are denoted as follows: *p < 0.05; **p < 0.01, determined by two-tailed unpaired Student’s *T*-tests; (**b**) A sample of the plates is shown, containing a set of cultures exposed to the media control extract (light gray bar) and a set exposed to the high-density wild type extract (dark gray bar).

Because it appeared that depletion of both *gprC* and *gprD* caused the fungus to stay locked in a low-density development pattern, we hypothesized that these receptors may be required for transmitting a signal produced at high density. The extract from the spent medium of a high density culture induced a significant increase in conidiation in the wild type [16], so this extract and an extract from un-inoculated plates (media control) were applied to cultures of the *gprC* and *gprD* mutants (Figure 4). As previously reported, the high-density extract stimulated a significant increase in conidiation when applied to the wild type (*p* = 0.002). In contrast to wild type, conidiation for all three mutants appeared to be slightly decreased in response to the high-density extract, though this was only minimally significant for the *∆gprC* strain (*p* = 0.049). 

#### 2.3.2. *gprC* and *gprD* Mutants Respond to Linoleic Acid

**Figure 5:**
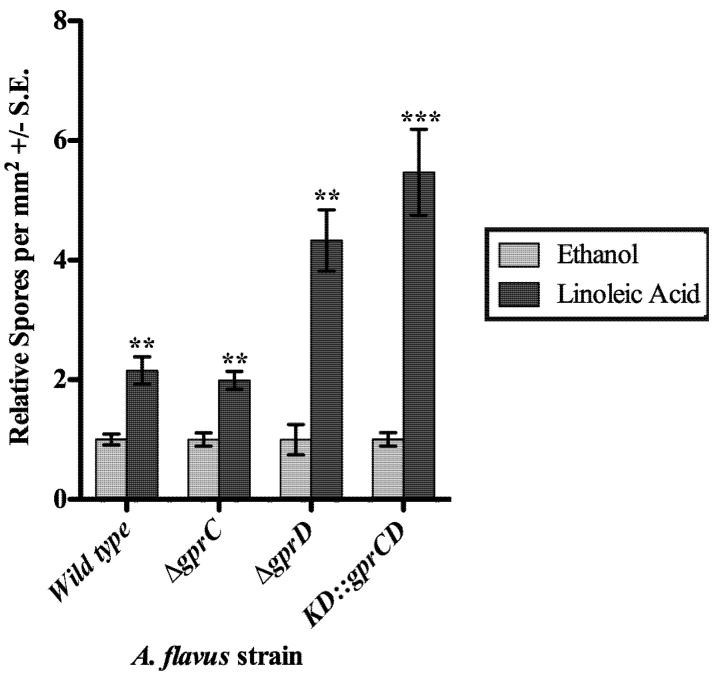
Spores were counted from the area of a plate surrounding a disk soaked with ethanol or linoleic acid in ethanol. The counts from the ethanol control disks were set to one, and the spore totals from the same strain exposed to linoleic acid (dark gray bars) were expressed relative to the ethanol control spore counts (light gray bars). Differences between the two treatments for each strain are denoted as follows: *p < 0.05; **p < 0.01; ***p < 0.001, determined by two-tailed unpaired Student’s *T*-tests.

Next the three mutants were assessed for their ability to respond to linoleic acid (LA), a known inducer of conidiation in *A. flavus* [29]. Conidiation was measured in response to disks containing 1 mg LA or an ethanol control (Figure 5). As previously reported, the wild type exhibited a significant increase in conidiation in response to LA (*p* = 0.003). In a similar fashion, all three mutants also produced significantly more conidia when exposed to LA (*p* = 0.002 for *∆gprC*, *p* = 0.001 for *∆gprD*, and *p* = 0.0008 for *KD::gprCD*). Although the relative increase in conidiation for the *KD::gprCD* strain from the ethanol control, 5.5-fold, was greater than that of any other strain tested, it still produced fewer conidia than any of the other strains (data not shown), as seen in the previous experiments. 

### 2.4. Discussion

This study has revealed several important findings. First of all, we have shown that *A. nidulans* responds to several plant oxylipins with a burst in cAMP in a dose-dependent manner (Figure 1 and Figure 2). Activation of a GPCR and subsequent activation and dissociation of the heterotrimeric G protein can lead to activation of adenylate cyclase, which converts ATP to cAMP. cAMP then catalyzes the release of protein kinase A (PKA) inhibitory subunits, resulting in activation of the kinase. Mutations made at various steps in this pathway in *A. nidulans* affect germination, sporulation, and secondary metabolism including mycotoxin synthesis [42,43,44,45,46]. These same developmental events are also greatly impacted in *A. nidulans* by applications of both endogenous and exogenous oxylipins [29], and our findings that oxylipins stimulate accumulation of cAMP provides evidence that oxylipins could be ligands initiating the PKA signaling pathway. Furthermore, the *A. nidulans ∆gprD* mutant did not exhibit a cAMP burst in response to plant oxylipins (Figure 2), suggesting that GprD may be an oxylipin receptor. Although no fungal GPCR has yet been identified as an oxylipin receptor, several classes of mammalian GPCRs sense a variety of oxylipins, including prostaglandins, leukotrienes, and the 9(*S*)-HpODE and 13(*S*)-HpODE oxylipins and their derivatives [30,31,36].

*In silico* analysis of GprD identified two putative homologs in *A. flavus*, GprC and GprD (Figure S2). Assessment of conidial and sclerotial production of individual deletion mutants as well as the double mutant *KD::gprCD* compared with the wild type demonstrate that GprC and GprD share overlapping functions in regulating density-dependent development (Figure 3). In every case, the differences between the wild type and the *KD::gprCD* strain were at least as—or often more—severe than those observed between the single mutants and wild type. Overall, the most striking differences between the wild type and the mutants occurred when grown at high-density. High density cultures of the *KD::gprCD* strain produced low amounts of conidia and high amounts of sclerotia and AF, mimicking a wild type low density culture. Moreover, the *KD::gprCD* mutant did not conidiate in response to spent media from a wild type high density culture (Figure 4), further suggesting that the loss of GprC and GprD locks the fungus into a low density state. Interestingly, this mutant was able to conidiate in response to linoleic acid (Figure 5), suggesting that the lack of response to the high density extract was signal-specific, and not due to a general conidiation defect. 

**Figure 6:**
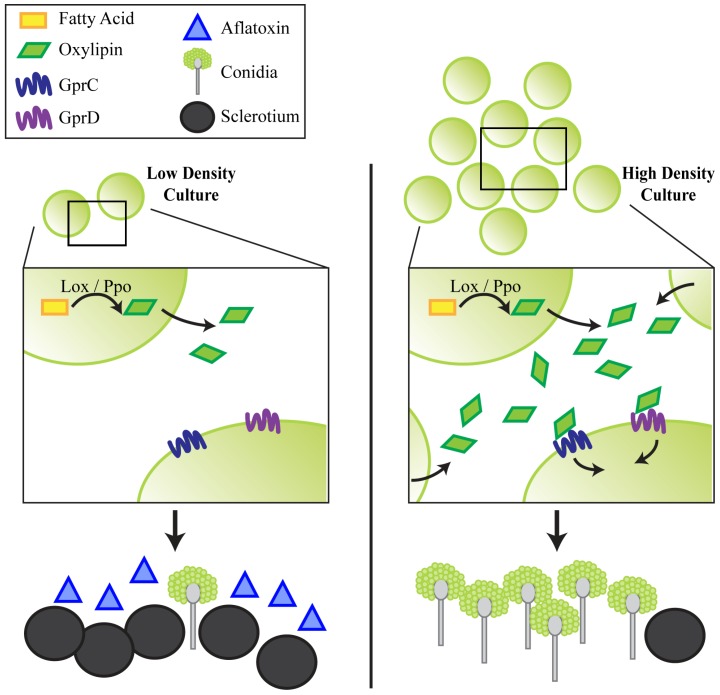
A hypothetical model is presented in which at low density, cultures of *A. flavus* produce low amounts of oxylipins (via Ppo and/or Lox enzymes). GprC/D signaling is not activated, and the culture produces AF and sclerotia, but very few conidia. At high density, more oxylipins are produced until their levels exceed a threshold and are recognized by GprC and GprD. This initiates a developmental shift toward conidiation, while very low amounts of sclerotia and AF are produced.

The phenotypes of the *gpr* mutants recapitulated those observed for several of the *A. flavus* oxygenase mutants, in particular strains deleted for the sole lipoxygenase gene, *loxA* and one of the *ppo* genes, *ppoC* [16,17]. Loss of either one of these genes similarly locked the fungus into a low density phenotype. Like that of the *KD::gprCD* mutant, the severity of the phenotype was additive in *A. flavus* strains depleted for the *lox* and multiple *ppo* genes. Considering these data together, we present a hypothetical model for GprC/D perception of endogenous oxylipins (Figure 6). We propose that at high-densities, a QS signal triggers wild type cultures to make very little AF and few sclerotia while conidiating profusely. GprC and GprD could be direct receptors of this signal(s), which could be generated by Lox and/or Ppo enzymes. Without these receptors or the enzymes that produce the signals, the fungus remains in a low-density state. Endogenous oxylipins are likely similar in structure to exogenous, plant derived oxylipins, and thus GprC and GprD could be important for fungal-host interactions.

## 3. Experimental Section

### 3.1. Culture Conditions

Strains (Table 1) were grown on glucose minimal medium (GMM) (6.0 g NaNO_3_, 0.52 g KCl, 0.52 g MgSO_4_·7H_2_O, 1.52 g KH_2_PO_4_, 1 mL trace elements stock solution (2.2 g ZnSO_4_·7H_2_O, 1.1 g H_3_BO_3_, 0.5 g MnCl_2_·4H_2_O, 0.5 g FeSO_4_·7H_2_O, 0.16 g CoCl_2_·5H_2_O, 0.16 g CuSO_4_·5H_2_O, 0.11 g (NH_4_)_6_Mo_7_O_24_·4H_2_O, 5.0 g Na_4_EDTA in 100 mL distilled H_2_O), 10 g glucose, 15 g agar, pH 6.5, in 1 L distilled H_2_O) unless mentioned otherwise. RKIS1 and RKIS47.1 were grown in medium supplemented with 0.001% para-amino benzoic acid. We forced sporulation of the *ΔcyaA* mutant RKIS47.1 by amending GMM with 0.8 M sorbitol; for uniformity, in the experiment depicted in Figure S1, spores for both RKIS1 and RKIS47.1 were generated on GMM + 0.8 M sorbitol. For genomic DNA extraction, strains were grown on liquid GMM with 0.5% yeast extract added. 

### 3.2. Strain Construction

Strain genotypes and sources are summarized in Table 1. Here, we describe in detail the construction of the *A. flavus* strains generated for this study. All primers are listed in Table 2*. gprC* and *gprD* were disrupted using homologous recombination to replace the gene with *pyrG* in the parental strain 3357-5 [51] (Figure S1a). The *gprC* 5' and 3' flanks were amplified with primers 1 and 2 and primers 3 and 4, respectively. The *gprD* 5' and 3' flanks were amplified with primers 5 and 6 and primers 7 and 8, respectively. *A. fumigatus pyrG* was amplified from genomic DNA using primers 9 and 10, and *A. parasiticus pyrG* was amplified from the plasmid pJMP7 using primers 11 and 12. Primers 1 and 4 and primers 5 and 8 were used to amplify the entire deletion constructs for *gprC* and *gprD*, respectively. 

To deplete both *gprC* and *gprD* transcripts in one strain using RNAi, the strategy laid out in McDonald *et al*. [52] was employed (Figure S1b), briefly described here: primers 13 and 14 and primers 15 and 16 were used to amplify ~500 bp of *gprC* and *gprD* coding regions, respectively. A single-joint PCR reaction was carried out with primers 13 and 16 to join the two fragments together and attach the appropriate restriction sites. The fragment was digested with *Asc*I and *Nco*I and ligated into pTMH44.2 [52] to form pKJA23. The same fragment was then digested with *Bam*HI and *Hin*dIII and ligated into pKJA23 to form pKJA26, containing inverted repeats with a Green Fluorescent Protein (GFP) spacer in between. *pyrG* was cut from pJW66.3 [22] using *Eco*RI and ligated into pKJA26 to form the final plasmid to be used for transformation into 3357-5 [51], pKJA27. 

**Table 2:** All oligonucleotide primers used in this study.

No.	Name	Sequence (5' to 3')
*1*	KS AflgprC 5'fk F	ttcctgcggcggttcattcc
*2*	KS AflgprC 5'fk+AfupyrG R	cgaagagggtgaagagcattgtttgaggcagtataagccagtcgtcgtgc
*3*	KS AfupyrG+AflgprC 3'fk F	gtgacgacaatacctcccgacgatacctggagtgaccggtcgagcaaagg
*4*	KS AflgprC 3'fk R	agcggttaagttctgtgtcc
*5*	KS Afl gprD 5'fk F	tcatatatccagtcccagtc
*6*	KS Afl gprD+AppyrG 5'fk R	ctcgggccatctgttcgtataagctttgttcatctcttgaggtgg
*7*	KS AppyrG+Afl gprD 3'fk F	agatccataggatcagcttatcgatgagtgtaccaggagactacg
*8*	KS Afl gprD 3'fk R	tgagacggatgtatggcttg
*9*	KS Ap pyrG pJMP7 F	agcttatacgaacagatggc
*10*	KS Ap pyrG pJMP7 R	tcgataagctgatcctatgg
*11*	KS Afu pyrG F	tgcctcaaacaatgctcttc
*12*	KS Afu pyrG R	ccaggtatcgtcgggaggt
*13*	KS H-N-AflgprD F	tataaagcttccatgggcttaatggtgaggatgctc
*14*	KS AflgprD-gprC R2	catgatggcgtaaaggcagacagtcccgaatcctagcacgataatgaagg
*15*	KS AflgprD-gprC F2	ggactatgggccttcattatcgtgctaggattcgggactgtctgcctttac
*16*	KS AflgprC-A-B R2	tataggatccggcgcgccatcgtcgtcttcacggtgtc
*17*	KS AflgprD2 ck F	gcttgacctggaaactttgc
*18*	KS AflgprD2 ck R	gatgatacggagacagaatg
*19*	KS JP M13 F	gtaaaacgacggccagtg
*20*	KS JP M13 R	ggaaacagctatgaccatg
*21*	KS AflgprC int F	cctttaccttcagcccaacg
*22*	KS AflgprC int R	atcagtccgagtgtgcttgc
*23*	KS AflgprD int F	gcttaatggtgaggatgctc
*24*	KS AflgprD int R	tgcgttcgagttggagtaag

Transformation of the fungus was carried out according to the protocol of Szewczyk *et al*. [53] with the following modifications: 200 mg of *Trichoderma* lysing enzymes (Sigma Aldrich, St. Louis, MO, USA) were added to 10 mL KCl protoplasting solution, and the protoplasts were plated on sorbitol minimal medium (SMM) (GMM plus 1.2 M sorbitol). All strains were confirmed by PCR (data not shown) and Southern analysis (Figure S3c-e), and in the case of the *IRTgprCD* strain, Northern analysis as well (Figure S3f). The primers used for the *∆gprC* and *∆gprD* Southern probes are primers 17 and 18 and primers 5 and 8, respectively. The primers used for the *IRTgprCD* Southern probe are primers 19 and 20, which amplify the *A. nidulans gpdA* promoter found in pTMH44.2. The primers used for the *IRTgprCD* Northern probes are primers 21 and 22 and primers 23 and 24. Samples for Northern blotting were grown according to these conditions: 10^6^ spores/mL in 50 mL liquid GMM in a 125-mL flask, with shaking at 30 °C at 250 r.p.m for 24 h. RNA was extracted using Isol-RNA Lysis Reagent (5 PRIME, Gaithersburg, MD, USA). 

### 3.3. cAMP Quantification

#### 3.3.1. Culture Conditions

10^6^ spores/mL were washed in GMM and suspended in 400 mL GMM in 1 L flasks, and grown at 37 °C at 300 r.p.m. for 18 h. At this time, mycelia were harvested by vacuum filtration, washed once with sterile GMM, and divided into 200 mg pieces which were inoculated into 20 mL fresh, sterile GMM in 50 mL conical tubes. These subcultures were equilibrated for 1 h at 37 °C at 300 r.p.m. 

Initially, different phenological stages of *A. nidulans* were compared for their response to 13(*S*)-HpODE: (*i*) 12-h and (*ii*) 18-h liquid shake cultures (cultured as described above), and (*iii*) tissues harvested from 18-h liquid shake cultures by filtration and asexually induced by spreading onto GMM agar and incubating for 4 h at 37 °C in constant light. Eighteen hours liquid shake cultures were the only ones in which 13(*S*)-HpODE treatment produced a significant change in cAMP concentrations (data not shown), so these conditions were used for all subsequent experiments. Depending on the experiment, three or four biological replicates were performed for each treatment. Each experiment was performed twice with similar results. 

#### 3.3.2. Treatment

After equilibration, cultures were treated with 12.5 μL of 5 mg/mL pure oxylipin (in EtOH) or with the same volume of pure EtOH as a control. Thus, oxylipins were at a final concentration of 10 μM. When other concentrations of oxylipins were needed, concentrated or diluted stocks were used so that the same volume, 12.5 μL (in EtOH), was always added to each sample. Samples were shaken by hand for precisely twenty seconds. Next, mycelia was separated from growth medium by filtration using Grade 1 Whatman filter paper (11 μM), scraped into microcentrifuge tubes, and flash-frozen in liquid nitrogen. Samples were stored for a maximum of 24 h at −80 °C until extraction and cAMP quantification.

#### 3.3.3. Extraction

Frozen mycelia were ground to a fine powder with a clean, pre-chilled mortar and pestle in liquid nitrogen and weighed on a microbalance. Tissue was resuspended by vortexing in 50 mM Tris.HCl/4 mM EDTA (pH 7.5) followed by boiling for five minutes and subsequent acidification by addition of concentrated HCl to a final concentration of 0.1 M. Samples were then incubated for ten minutes on ice and centrifuged for five minutes at 13,000 r.p.m. at 4 °C. The supernatant was transferred to a fresh microcentrifuge tube and flash-frozen in liquid nitrogen. Acidified supernatants were split into two subsamples, one for quantification of cAMP and the other for quantification of protein. 

#### 3.3.4. Quantification of cAMP

cAMP was measured using the Direct cAMP colorimetric (EIA) kit (Enzo Life Sciences, Exeter, UK) according to the manufacturer’s directions. Reactions were performed in 96 well plates and optical density (OD_405_–OD_595_) was measured using a Victor^3^ V multiwell plate reader (Perkin Elmer, Waltham, MA, USA). Corrected absorbance readings were fitted to a four-parameter sigmoidal model, and cAMP concentrations of unknowns from each plate were interpolated from that individual plate’s standard curve. The *R*^2^ for all plate-specific standard curves was always ≥0.99 except for the *∆cyaA* test plate, for which the *R*^2^ was 0.94. Two technical replications were performed on each sample.

#### 3.3.5. Protein Quantification

Protein content was measured using the Bradford reagent supplied by BioRad (Hercules, CA, USA) according to the manufacturer’s directions. To neutralize the 0.1 M HCl in the samples to be used for protein quantification, an equal volume of 0.1 N NaOH was added to each subsample.

### 3.4. Oxylipins and Fatty Acids

13(*S*)-Hydroperoxy-*cis*-9,*trans*-11-octadecadienoic acid [13(*S*)-HpODE], 9(*S*)-hydroperoxy-*trans*-10,*cis*-12-octadecadienoicacid [9(*S*)-HpODE], 9(*S*)-hydroxy-*trans*-10,*cis*-12-octadecadienoic acid [9(*S*)-HODE], 13(*S*)-hydroxy-*cis*-9,*trans*-11-octadecadienoic acid [13(*S*)-HODE] and linoleic acid were purchased from Cayman Chemical (Ann Arbor, MI, USA).

### 3.5. Density-Dependent Physiology

Strains were grown and analyzed as described in Horowitz Brown *et al*. [16]. Sixty millimeters diameter plates containing 10 mL GMM + 2% sorbitol (1.5% agar) were overlaid with 3 mL GMM + 2% sorbitol (0.75% agar) containing 10^3^ or 10^7^ spores. Eight replicates per set were placed in the dark at 29 °C for seven days. Four replicates were used for sclerotia quantification, and the other four replicates were used for enumeration of conidia and AF extraction. For sclerotia analysis, plates were sprayed with 70% ethanol to remove conidia, and sclerotia were scraped off with a spatula. The sclerotia were placed into pre-weighed microcentrifuge tubes, flash-frozen in liquid nitrogen, lyophilized, and weighed. Three 15-mm diameter cores were removed from each of the other four replicates and homogenized in 4.5 mL 0.01% Tween H_2_O. A 100-μL aliquot was removed from each tube, diluted, and the spores were counted on a hemacytometer. To extract AF, 3 mL of chloroform were added to each tube, the tubes were shaken vigorously and spun at 3000 r.p.m. for 10 min. The chloroform layer was removed, dried down, and resuspended in 100 μL chloroform. 10 μL was spotted onto a thin layer chromatography (TLC) plate alongside an AFB_1_ standard, and the plates were placed in a TLC chamber containing 9:1 chloroform:acetone. After separation of metabolites, the plates were visualized at 366 nm, and the spot intensities were measured using Adobe Photoshop (version 11.0; Adobe Systems Incorporated: San Jose, California, USA, 2008). Absolute intensity was calculated by multiplying the mean pixel intensity by the number of pixels in the selected area encompassing an AF spot.

### 3.6. Spent Media Bioassay

#### 3.6.1. Extract Collection

Extracts from high-density and media-only plates were collected as described [16]. Briefly, thirty 100-mm diameter plates containing 25 mL GMM + 2% sorbitol (1.5% agar) were overlaid with 10 mL GMM + 2% sorbitol (0.75% agar) containing 10^7^*A. flavus* wild type (NRRL3357) spores. The same set-up was repeated with no spores added for a media-only control. All plates were incubated in the dark at 29 °C for five days. The plate contents were homogenized in a blender (ten plates plus 250 mL sterile distilled H_2_O a time) and transferred to a 4-L beaker. One volume of ethyl acetate was mixed in, and the macerate was allowed to settle overnight. The organic layer was removed, filtered through Whatman™ #1 filter paper and evaporated using a rotary evaporator. The final extract was resuspended in 2.5 mL ethanol, topped with nitrogen gas, and stored at −80 °C. 

#### 3.6.2. Bioassay

The extracts were diluted 1:10 in ethanol, and 100 μL of diluted extract was spread onto 60-mm diameter plates containing 10 mL GMM + 2% sorbitol (1.5% agar). Plates were allowed to dry for fifteen minutes and were then inoculated with 10^6^ spores in 3 mL GMM + 2% sorbitol (0.75% agar). Four replicates per strain per treatment were prepared and placed in the dark at 29 °C for three days. Enumeration of conidia was carried out as described for density-dependent physiology tests.

### 3.7. Linoleic Acid Disk Assay

Sixty-millimeter diameter plates containing 10 mL GMM + 2% sorbitol (1.5% agar) were overlaid with 10^5^ spores in 3 mL GMM + 2% sorbitol (0.75% agar). Linoleic acid in ethanol (1 mg per disk in a 15 μL volume) was applied to sterile 12.7 mm diameter filter disks. Control disks were prepared with 15 μL ethanol. Once the disks dried, one disk was applied to the center of each plate for a total of four replicates per treatment per strain. The plates were placed in the dark at 29 °C for six days. Following this incubation, a 14-mm core containing the filter disk was removed and discarded. A 22-mm core, with the removed portion directly centered, was punched out, and this ring was homogenized in 3 mL 0.01% Tween H_2_O. Spore enumeration continued from this point as previously described.

### 3.8. Statistical Analysis

In Figure 1 and Figure 2, one-tailed paired Student’s *T*-tests were performed using the T.TEST function in Microsoft Excel in the Microsoft Office Professional Plus 2010 package. In Figure 1B, treatment means were compared using a Student’s *T*-tests following an analysis of variance (single-factor ANOVA; MS Excel). In Figure 3, ANOVA was performed with Prism 5^®^ software using one-way ANOVA and Tukey’s multiple comparison post-test. In Figure 4 and Figure 5, two-tailed unpaired Student’s *T*-tests were performed using the T.TEST function in Microsoft Excel in the Microsoft Office 2008 package.

## 4. Conclusions

We found that GprD was essential for the induction of cAMP in *A. nidulans* in response to exogenous plant oxylipins. We also found that GprC and GprD in *A. flavus* were essential for the perception of a still-unknown signal found in high-density cultures that induces sclerotia formation while inhibiting AF and conidial formation. The finding of fungal receptors critical for *A. flavus* development and potentially receptive to seed signals is an exciting prospect for future study. Not only does the identity of such receptors deepen our knowledge of fungal biology but also carries great potential for combatting pathogenic fungi. Mammalian GPCRs are extensively studied for their therapeutic potential—approximately half of all drugs target GPCRs [54], and so drugs that can block critical fungal GPCRs may serve as valuable anti-fungal treatments.

## Supplementary
